# Worldwide diversity of endophytic fungi and insects associated with dormant tree twigs

**DOI:** 10.1038/s41597-022-01162-3

**Published:** 2022-03-01

**Authors:** Iva Franić, Simone Prospero, Kalev Adamson, Eric Allan, Fabio Attorre, Marie Anne Auger-Rozenberg, Sylvie Augustin, Dimitrios Avtzis, Wim Baert, Marek Barta, Kenneth Bauters, Amani Bellahirech, Piotr Boroń, Helena Bragança, Tereza Brestovanská, May Bente Brurberg, Treena Burgess, Daiva Burokienė, Michelle Cleary, Juan Corley, David R. Coyle, György Csóka, Karel Černý, Kateryna Davydenko, Maarten de Groot, Julio Javier Diez, H. Tuğba Doğmuş Lehtijärvi, Rein Drenkhan, Jacqueline Edwards, Mohammed Elsafy, Csaba Béla Eötvös, Roman Falko, Jianting Fan, Nina Feddern, Ágnes Fürjes-Mikó, Martin M. Gossner, Bartłomiej Grad, Martin Hartmann, Ludmila Havrdova, Miriam Kádasi Horáková, Markéta Hrabětová, Mathias Just Justesen, Magdalena Kacprzyk, Marc Kenis, Natalia Kirichenko, Marta Kovač, Volodymyr Kramarets, Nikola Lacković, Maria Victoria Lantschner, Jelena Lazarević, Marianna Leskiv, Hongmei Li, Corrie Lynne Madsen, Chris Malumphy, Dinka Matošević, Iryna Matsiakh, Tom W. May, Johan Meffert, Duccio Migliorini, Christo Nikolov, Richard O’Hanlon, Funda Oskay, Trudy Paap, Taras Parpan, Barbara Piškur, Hans Peter Ravn, John Richard, Anne Ronse, Alain Roques, Beat Ruffner, Karolis Sivickis, Carolina Soliani, Venche Talgø, Maria Tomoshevich, Anne Uimari, Michael Ulyshen, Anna Maria Vettraino, Caterina Villari, Yongjun Wang, Johanna Witzell, Milica Zlatković, René Eschen

**Affiliations:** 1 CABI, Delémont, Switzerland; 2 Swiss Federal Institute for Forest, Snow and Landscape Research WSL, Birmensdorf, Switzerland; 3 Institute of Plant Sciences, University of Bern, Bern, Switzerland; 4 Institute of Forestry and Rural Engineering, Estonian University of Life Sciences, Tartu, Estonia; 5 Department of Environmental Biology, Sapienza University of Rome, Rome, Italy; 6 Forest Zoology Research Unit, French National Research Institute for Agriculture, Food and Environment (URZF INRAE), Orléans, France; 7 Forest Research Institute, Hellenic Agricultural Organization – Demeter, Thessaloniki, Greece; 8 Meise Botanic Garden, Meise, Belgium; 9 Institute of Forest Ecology, Slovak Academy of Sciences, Nitra, Slovakia; 10 National Research Institute of Rural Engineering, Water and Forests (INRGREF), Ariana, Tunisia; 11 Department of Forest Ecosystems Protection, University of Agriculture in Krakow, Krakow, Poland; 12 Instituto Nacional de Investigação Agrária e Veterinária I. P. (INIAV I. P.), Oeiras, Portugal; 13 GREEN-IT Bioresources for Sustainability, ITQB NOVA, Oeiras, Portugal; 14 Silva Tarouca Research Institute for Landscape and Ornamental Gardening, Pruhonice, Czech Republic; 15 NIBIO, Norwegian Institute of Bioeconomy Research, Ås, Norway; 16 NMBU - Norwegian University of Life Sciences, Ås, Norway; 17 Harry Butler Institute, Murdoch University, Murdoch, Western Australia Australia; 18 Institute of Botany at the Nature Research Centre, Vilnius, Lithuania; 19 Southern Swedish Forest Research Centre, Swedish University of Agricultural Sciences, Alnarp, Sweden; 20 Instituto de Investigaciones Forestales y Agropecuarias Bariloche (INTA-CONICET), Bariloche, Argentina; 21 Department of Forestry and Environmental Conservation, Clemson University, Clemson, South Carolina USA; 22 University of Sopron, Forest Research Institute, Department of Forest Protection, Mátrafüred, Hungary; 23 Ukrainian Research Institute of Forestry and Forest Melioration, Kharkiv, Ukraine; 24 Slovenian Forestry Institute, Ljubljana, Slovenia; 25 Sustainable Forest Management Research Institute, University of Valladolid—INIA, Palencia, Spain; 26 Department of Vegetal Production and Forest Resources, University of Valladolid, Palencia, Spain; 27 Isparta University of Applied Sciences, Isparta, Turkey; 28 School of Applied Systems Biology, La Trobe University, Melbourne, Victoria Australia; 29 Agriculture Victoria Research, Agribio Centre, Bundoora, Victoria Australia; 30 Ukrainian Research Institute of Mountain Forestry, Ivano-Frankivsk, Ukraine; 31 College of Forestry and Biotechnology, Zhejiang A & F University, Hangzhou, China; 32 Institute of Terrestrial Ecosystems, ETH Zürich, Zürich, Switzerland; 33 Institute of Agricultural Sciences, ETH Zürich, Zürich, Switzerland; 34 Department of Geosciences and Natural Resource Management, University of Copenhagen, Copenhagen, Denmark; 35 Sukachev Institute of Forest, Siberian Branch of the Russian Academy of Sciences, Federal Research Center “Krasnoyarsk Science Center SB RAS”, Krasnoyarsk, Russia; 36 Siberian Federal University, Krasnoyarsk, Russia; 37 Croatian Forest Research Institute, Jastrebarsko, Croatia; 38 Ukrainian National Forestry University, Lviv, Ukraine; 39 Biotechnical Faculty, University of Montenegro, Podgorica, Montenegro; 40 CABI, Beijing, China; 41 Fera Science Ltd, National Agri‐food Innovation Campus, York, UK; 42 Royal Botanic Gardens Victoria, Melbourne, Victoria Australia; 43 National Plant Protection Organisation, Netherlands Food and Consumers Product Safety Authority, Ministry of Agriculture, Nature and Food Quality, Wageningen, Netherlands; 44 Institute for Sustainable Plant Protection (IPSP), National Research Council C.N.R., Sesto Fiorentino, Italy; 45 National Forest Centre, Forest Research Institute, Zvolen, Slovakia; 46 Present Address: Department of Agriculture, Food and the Marine, Dublin, Republic of Ireland; 47 Agri-Food & Biosciences Institute (AFBI), Belfast, UK; 48 Faculty of Forestry, Çankırı Karatekin University, Cankiri, Turkey; 49 Forestry and Agricultural Biotechnology Institute (FABI), University of Pretoria, Pretoria, South Africa; 50 Tanzania Forestry Research Institute (TAFORI), Lushoto, Tanzania; 51 Central Siberian Botanical Garden, Russian Academy of Sciences, Siberian Branch, Novosibirsk, Russia; 52 Natural Resources Institute Finland, Suonenjoki, Finland; 53 USDA Forest Service, Southern Research Station, Athens, Georgia USA; 54 DIBAF, University of Tuscia, Viterbo, Italy; 55 D.B. Warnell School of Forestry & Natural Resources, University of Georgia, Athens, Georgia USA; 56 Forestry and Wood Technology, Linnaeus University, Växjö, Sweden; 57 Institute of Lowland Forestry and Environment (ILFE), University of Novi Sad, Novi Sad, Serbia; 58 South African National Biodiversity Institute, Kirstenbosch Research Centre, Cape Town, South Africa

**Keywords:** Biodiversity, Forest ecology, Biogeography, Community ecology

## Abstract

International trade in plants and climate change are two of the main factors causing damaging tree pests (i.e. fungi and insects) to spread into new areas. To mitigate these risks, a large-scale assessment of tree-associated fungi and insects is needed. We present records of endophytic fungi and insects in twigs of 17 angiosperm and gymnosperm genera, from 51 locations in 32 countries worldwide. Endophytic fungi were characterized by high-throughput sequencing of 352 samples from 145 tree species in 28 countries. Insects were reared from 227 samples of 109 tree species in 18 countries and sorted into taxonomic orders and feeding guilds. Herbivorous insects were grouped into morphospecies and were identified using molecular and morphological approaches. This dataset reveals the diversity of tree-associated taxa, as it contains 12,721 fungal Amplicon Sequence Variants and 208 herbivorous insect morphospecies, sampled across broad geographic and climatic gradients and for many tree species. This dataset will facilitate applied and fundamental studies on the distribution of fungal endophytes and insects in trees.

## Abstract (summary)

**Table:** 

Measurement(s)	metagenomics analysis • Cytochrome C Oxidase Subunit 1
Technology Type(s)	amplicon sequencing • Dideoxy Chain Termination DNA Sequencing
Factor Type(s)	tree species • geographic location • mean annual temperature • mean annual precipitation
Sample Characteristic - Organism	Fungi • Insecta
Sample Characteristic - Environment	dormant tree twigs

Machine-accessible metadata file describing the reported data: 10.6084/m9.figshare.16764229

## Background & Summary

Fungi and insects can have large impacts on tree health, ranging from beneficial to very harmful^1–3^. In the last 200 years the number and impact of tree pests (i.e. harmful fungi and insects) has considerably increased^4–6^, mainly because non-native pests have been introduced to new areas through the global trade of plant material^4,7^. However, our current knowledge about the distribution of organisms associated with trees comes from studies that explored the diversity of fungi and insects at local and regional scales^8–11^. These studies rarely focused on more than one taxon^12^, or sampled multiple hosts in different geographic regions^12–15^. The general lack of large-scale biodiversity studies is mainly due to a lack of resources, as surveys can be laborious, costly, and often require strong collaborative networks^16^. Large-scale biodiversity assessments have only been done for a few organism groups, including soil fungi^17^, earthworms^18^, plants^19^ and terrestrial vertebrates^20^. Taxa associated with a single host species or genus have never been investigated in a standardized study across several continents, although this would be valuable to test general hypotheses related to global biodiversity patterns and to assess the risk associated with the global trade of plants.

**Fig. 1:**
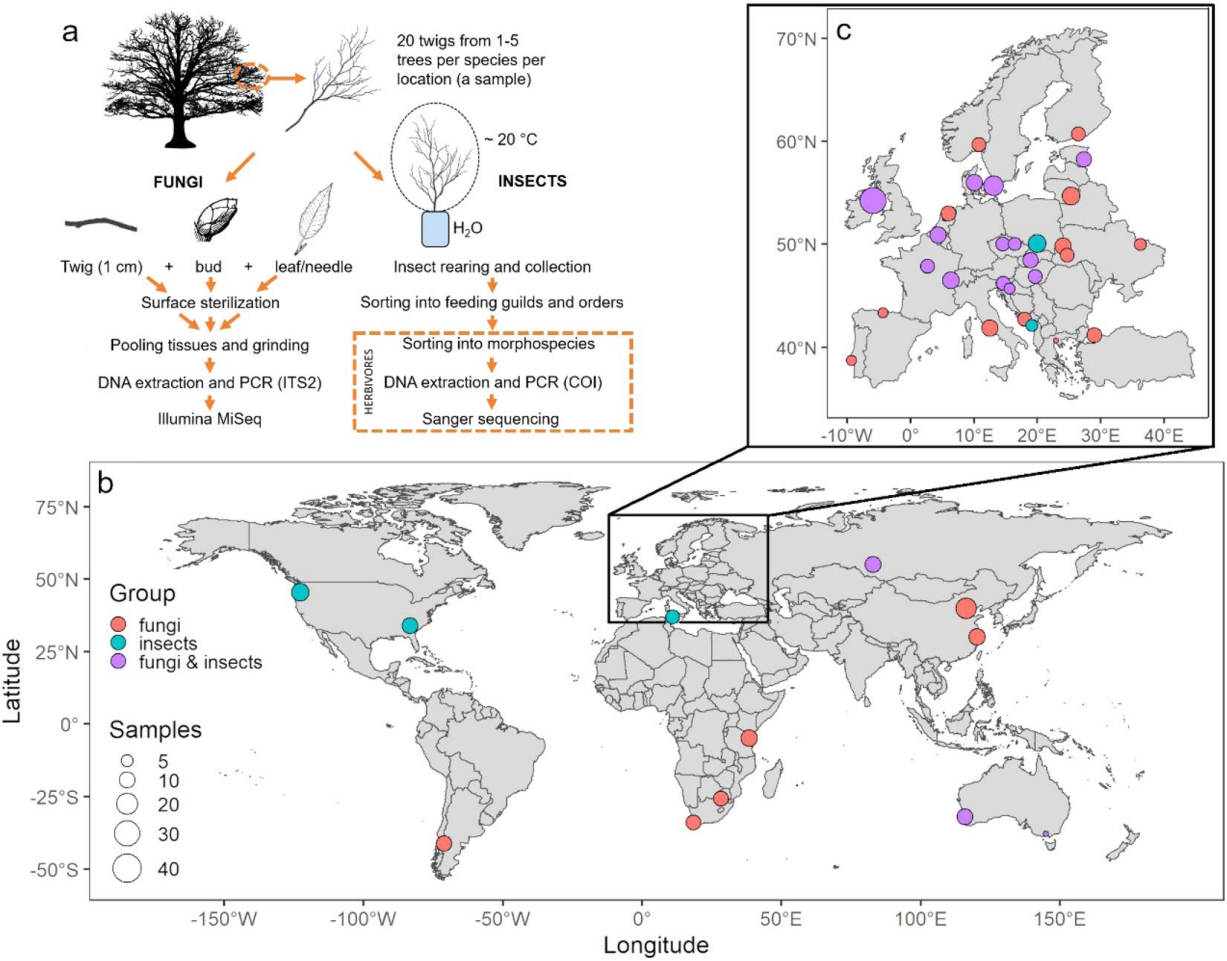
Description of sample collection and sampling locations for the study. (**a**) Twenty twigs per tree were collected from 1–5 trees per species at each location. One sample constitutes all twigs from a given tree species at a particular location. Fungi were assessed by high-throughput sequencing from 50 mg of surface sterilized and ground tissues (i.e. pooled 0.5 cm long twig segments, halves of buds, and 0.5 cm long needle/leaf segments for evergreen species collected from a sample). Insects were reared from collected twigs and identified using morphological and molecular approaches. (**b,c**) Sampling locations outside Europe (**b**) and within Europe (**c**) are shown. Colors indicate the groups that were assessed from collected samples. The size of the circles is proportional to the number of samples collected at each location. The same legend applies for b and c. In several cases, samples collected at multiple locations within a country were merged for better visibility (i.e. AR, AU, EE, HU, ME, SE, TN, UK, ZA; Table 1).

**Fig. 2:**
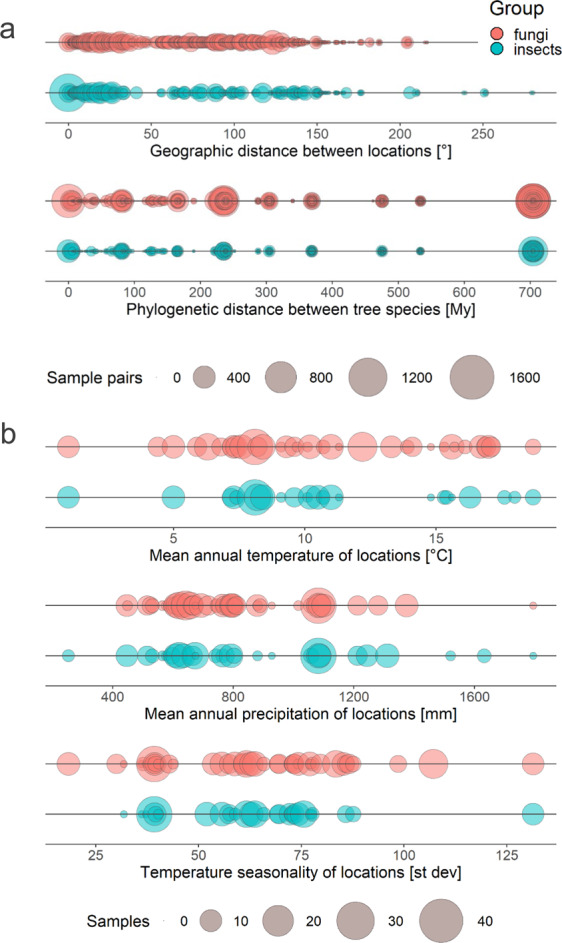
Distribution of collected samples along gradients of different variables. (**a**) Frequency of values for pairwise comparisons of host geographic origin (geographic distance) and host species (phylogenetic distance). (**b**) Frequency distribution of climate variables for sites (i.e. mean annual temperature, temperature seasonality and mean annual precipitation). The size of the circles indicates the number of sample pairs (**a**) or samples (**b**). Colors indicate different groups. Geographic distance is calculated as Euclidean distance and is expressed in degrees (°) with 1° corresponding to 111 km. Phylogenetic distance is expressed in millions of years (My).

We studied overwintering stages of endophytic fungi and insects associated with the twigs of multiple tree species, collected around the globe, during the winter of 2017/2018 (Fig. 1). This was done through the COST Action FP1401 “Global warning”, which aimed to create standardized protocols for the establishment and monitoring of sentinel plantings^21^ on a global scale, as an early warning system to detect potential tree pests. Specifically, this data set was compiled to determine which pests might be moved through trade of asymptomatic plant material. We sampled dormant twigs on multiple trees: 20 twigs were sampled per tree species and location (“a sample”). In total 145 native and non-native tree species, both angiosperms and gymnosperms, were sampled in 28 countries (total of 352 samples). Fungi were assessed by high-throughput sequencing (HTS). Insects were reared from dormant twigs collected from a subset of 109 tree species in 18 countries (total of 227 samples) and were sorted to taxonomic orders and feeding guilds. Herbivorous insects were identified by morphological and molecular methods. This data set reveals the diversity of tree-associated fungal endophytes and insects across broad geographic and climatic gradients and for many host taxa (Fig. 2). The data set can be used to investigate the biodiversity of tree-associated fungal endophytes and insects, especially in studies comparing different geographic and climatic regions and different hosts. Furthermore, the results of such analyses can be used in Pest/Pathway Risk Analyses, with the ultimate goal of reducing the likelihood of pest introductions to new areas.

The data set includes raw sequence data obtained from HTS of the internal transcribed spacer region 2 (ITS2) of the ribosomal operon from dormant twig tissues^22^. We also present a sample x Amplicon Sequence Variant (ASV^23^) matrix, which consists of abundances of 12,721 fungal ASVs in 352 samples^24^ and data on the taxonomic classification of detected fungal ASVs^24^. We present similar data for insects: specimen counts for insects belonging to different feeding guilds (i.e. herbivores, predators and parasitoids) and taxonomic orders in each sample^24^ and a sample x morphospecies matrix^24^ showing abundances of 208 herbivorous insect morphospecies across 227 collected samples, together with data on the tentative identification of herbivorous insect morphospecies^24^. Aligned cytochrome c oxidase subunit 1 (COI) sequences of herbivorous insects are deposited in GenBank^25^. Finally, we provide a table containing information about tree species, location and climate for each sample^24^.

## Methods

### Field collection

Endophytic fungi and insects were assessed from dormant twig samples from 155 tree species at 51 locations in 32 countries. Sampled tree species belonged to genera that are native to, and occur widely across, either the northern or southern hemisphere, since very few tree genera occur naturally in both hemispheres (e.g., in our study only *Podocarpus* appears in both hemispheres but has a limited distribution in the northern hemisphere). We sampled largely in botanical gardens and arboreta, which allowed us to sample native and non-native, congeneric and confamiliar, tree species at each location. At each location, one native and one to three non-native congeneric or confamiliar tree species were sampled.

At each location, twenty 50-cm long asymptomatic twigs were collected from 1–5 individual trees per species, from different branches and different parts of the crown (Fig. 1). The number of individual trees per species depended on the number of trees available in the specific botanical garden or arboretum, which was often low (Table 1). All twigs per tree species and location were pooled and analysed as a single sample. On some occasions two samples of the same tree species at the same location are considered. Sampling was conducted in the month with the shortest day-length in the year (end of December 2017 in the Northern hemisphere, end of June 2018 in the Southern hemisphere). Samples originating from a tropical region (eleven samples from Tanzania) were collected in June 2018. Trees were sampled in winter to align with the timing of trade, i.e. most woody plants are traded in winter or early spring, as plants will be planted in the following spring, and to reduce the risk of introducing foliar pests in deciduous trees. Evergreen gymnosperm and angiosperm tree species, which were also considered, do not lose foliage during winter, and are thus sold with leaves/needles.

**Table 1:** Site information for sampling locations included in this study.

Continent	Country	Location	Lon	Lat	Group	Trees per sample (N)	Samples (N)	Tree genera (N)	Ann Temp (°C)	Temp Season (st dev)	Ann Prec (mm)
Africa	South Africa	Capetown	18,46	−33,99	F	3,9	8	6	16,9	30,07	791
Africa	South Africa	Pretoria	28,23	−25,75	F	4,1	8	6	17,1	39,33	717
Africa	Tanzania	Lushoto	38,30	−4,79	F	5,5	11	5	17	18,36	1098
Africa	Tunisia	Dar Chichou	10,96	36,97	I		3	3	18	58,94	254
Africa	Tunisia	Sajanan	9,24	37,06	I		4	3	17,6	57,54	742
Asia	China	Beijing	116,21	40,00	F	1,2	18	5	12,2	107,17	642
Asia	China	Hangzhou	120,12	30,25	F	1,4	11	5	16,7	85,27	1375
Asia	Russia	Novosibirsk	82,88	55,06	F & I	2,3	10	5	1	131,43	448
Australia	Australia	Nannup	115,77	−33,98	F & I	5,0	1	1	15,6	36,14	928
Australia	Australia	Balingup	115,98	−33,79	F & I	1,0	2	1	15,3	40,21	881
Australia	Australia	Beech Forest	143,57	−38,64	F & I	5,0	1	1	11,3	31,8	1795
Australia	Australia	Melbourne	144,96	−37,82	F & I	5,0	1	1	14,8	37,23	675
Australia	Australia	Perth	115,85	−31,95	F & I	4,0	5	3	18,7	39,55	803
Europe	Belgium	Meise	4,33	50,93	F & I	2,3	12	6	10,2	55,7	793
Europe	Croatia	Dubrovnik	17,97	42,71	F		8	4	14,1	60,81	1281
Europe	Croatia	Jastrebarsko	15,64	45,67	F		3	2	11	72,68	1059
Europe	Croatia	Jastrebarsko	15,64	45,67	F & I		2	2	11	72,68	1059
Europe	Czech Republic	Průhonice	14,56	50,00	F & I	1,0	8	2	8,7	69,6	515
Europe	Czech Republic	Žampach	16,43	50,04	F & I	1,6	7	3	7,2	69,41	609
Europe	Denmark	Hørsholm	12,51	55,88	F & I	2,7	12	4	8,1	62,85	636
Europe	Estonia	Järvselja	27,33	58,25	F & I	3,8	5	2	5	87,64	609
Europe	Estonia	Tartu	26,73	58,38	F & I	3,7	6	2	5	85,81	597
Europe	Finland	Kouvola	26,42	60,73	F	4,3	8	2	4,4	86,66	616
Europe	France	Nogent-sur-Vernisson	2,74	47,85	F & I	3,4	8	2	11	57,38	666
Europe	Greece	Thessaloniki	22,94	40,64	F	2,0	2	2	15,7	74,22	449
Europe	Hungary	Gödöllő	19,36	47,60	F & I		2	1	10,1	77,19	566
Europe	Hungary	Kecskemét	19,69	46,89	F & I		4	1	10,7	77,67	532
Europe	Hungary	Mátrafüred	19,97	42,83	F & I		2	1	6,3	65,77	1128
Europe	Italy	Rome	12,47	41,89	F		12	5	15,6	58,84	797
Europe	Lithuania	Vilnius	25,28	54,69	F	1,0	15	3	6,3	83,45	657
Europe	Montenegro	Bar	19,09	42,09	I		2	2	15,3	63,7	1521
Europe	Montenegro	Podgorica	19,26	42,43	I		4	3	15,4	72,5	1632
Europe	Netherlands	Wageningen	5,31	52,03	F	3,1	10	4	9,3	53,58	796
Europe	Norway	Ås	10,79	59,69	F		8	3	5,9	73,2	773
Europe	Poland	Krakow	19,96	50,06	I		15	4	8,2	75,61	674
Europe	Portugal	Oeiras	−9,31	38,70	F	1,0	4	2	16,1	36,62	746
Europe	Slovakia	Banská Štiavnica	18,93	48,45	F & I	1,5	11	3	7,3	74,26	766
Europe	Slovenia	Volčji Potok	14,61	46,19	F & I	1,6	8	2	9,6	73,34	1213
Europe	Spain	Caviedes	−4,33	43,34	F	5,0	4	1	13,9	38,87	889
Europe	Sweden	Alnarp	13,08	55,66	F & I	1,6	15	4	8,1	61,68	621
Europe	Sweden	Göteborg	11,95	57,68	F & I	1,8	4	2	7,4	65,89	765
Europe	Switzerland	Aubonne	6,37	46,51	F & I	1,8	13	4	8,4	63,7	1084
Europe	Turkey	Istanbul	28,98	41,18	F	1,0	10	3	13,3	61,31	880
Europe	UK	Castelwellan	−5,94	54,26	F & I	1,0	27	8	8,7	39,2	1082
Europe	UK	Tollymore	−5,95	54,22	F & I	1,0	11	4	8,7	39,32	1086
Europe	Ukraine	Ivano-Frankivsk	24,71	48,92	F		8	3	8,2	79,68	669
Europe	Ukraine	Kharkiv	36,23	50,00	F		6	3	7,4	98,55	526
Europe	Ukraine	Lviv	24,03	49,84	F		12	3	7,6	77,11	693
North America	USA	Athens, Georgia	−83,38	33,90	I	1,0	10	5	16,3	72,27	1244
North America	USA	Portland, Oregon	−122,72	45,52	I	1,0	12	3	10,5	52,01	1311
South America	Argentina	Bariloche	−71,27	−41,19	F	1,5	7	6	6,8	42,83	812
South America	Argentina	El Bolsón	−71,54	−41,96	F		2	2	9,7	43,79	1017

For each sampling location geographic latitude and longitude are indicated, as well as the group that was sampled (insects and/or fungi are indicated with I and F, respectively). The table also shows the average number of individual trees per sample, number of collected samples, number of tree genera sampled at a location, and climatic variables (Ann Temp: mean annual temperature, Temp Season: temperature seasonality and Ann Prec: mean annual precipitation).

### Fungal endophytes

To assess fungal communities, a total of 352 samples from 145 native and non-native tree species, belonging to nine families of angiosperms and gymnosperms, were collected. Sampling was done at 44 locations in 28 countries on five continents (Fig. 1, Table 1).

From each twig in a sample, one bud, one needle/leaf and one 1 cm long twig segment were taken (Fig. 1). Needles from gymnosperms, and leaves from evergreen angiosperms were sampled to accurately assess the risk of trading these species. Twig segments were cut from the twig bases. The selected plant parts were surface sterilized by immersion in 75% ethanol for 1 min, 4% NaOCl for 5 min, and 75% ethanol for 30 s^26^. After air drying on a sterile bench, the following material from each of 20 twigs per sample was pooled: half of one bud, a 0.5 cm long piece of a needle (from gymnosperms) or a 0.25 cm^2^ leaf (for evergreen angiosperms) and a 0.5 cm long piece of twig.

#### DNA extraction, PCR amplification and Illumina sequencing

Total genomic DNA was extracted from 50 mg of pooled, surface sterilized, and ground tissue (Fig. 1) using DNeasy PowerPlant Pro Kit (Qiagen, Hilden, Germany), following the manufacturer’s instructions. For a total of 31 out of 352 samples, DNA was extracted from different tissues separately, and DNA extracts were then pooled. DNA concentrations were quantified using the Qubit dsDNA BR Assay Kit (Thermo Fisher Scientific, Waltham, USA) on a Qubit 3.0 Fluorometer (Thermo Fisher Scientific) and DNA was diluted to 5 ng/μl. Samples that yielded less than 5 ng/μl were not diluted. The ITS2 region was amplified with the 5.8S-Fung and ITS4-Fung primers^27^. PCR amplifications were carried out in 20 μl reaction volumes containing 25 ng of DNA template, 1 mg/ml BSA, 1 mM of MgCl_2_, 0.4 μM of each primer, and 0.76 × JumpStart REDTaq ReadyMix Reaction Mix (Sigma-Aldrich, Steinheim, Germany). PCR was performed using Veriti 96-Well Thermal Cycler (Applied Biosystems, Foster City, CA, USA) as described in Franić *et al*. (2019). Each sample was amplified in triplicates and successful PCR amplification confirmed by visualization of the PCR products, before and after pooling the triplicates, on 1.5% (w/v) agarose gel with ethidium bromide staining. Pooled amplicons were sent to the Génome Québec Innovation Center at McGill University (Montréal, Quebec, Canada) for barcoding using Fluidigm Access Array technology (Fluidigm, South San Francisco, CA, USA) and paired-end sequencing on the Illumina MiSeq v3 platform (Illumina Inc., San Diego, CA, USA). Raw sequences obtained in this study are deposited at the NCBI Sequence Read Archive under BioProject accession number PRJNA708148^22^.

#### Bioinformatics and taxonomical classification of ASVs

Quality filtering and delineation into ASVs were done with a customized pipeline^28^ largely based on VSEARCH^29^, as described by Herzog *et al*.^30^. The output data available on Figshare show the abundances of fungal ASVs in the samples^24^. Taxonomic classification of ASVs was conducted using Sintax^31^ implemented in VSEARCH against the UNITE v.7.2 database^32^ with a bootstrap support of 80%. The data on the taxonomic classification of fungal ASVs is deposited in Figshare^24^.

Quality filtering, delineation into ASVs, and taxonomical assignments were done on a larger data set (total of 474 samples), which increased the confidence in the selected centroid sequences. This data set consisted of (1) sequences obtained from 352 samples of pooled tree tissues that are presented here^22^, (2) sequences obtained from 33 samples of pooled tree tissues which were not included in this manuscript due to violation of the common protocol, (3) sequences from 21 contaminated samples (positive DNA extraction controls), including sequences from the two control samples (not presented here), and (4) sequences obtained from 66 samples of non-pooled tree tissues of *Pinus sylvestris* and *Quercus robur* that were collected from the subset of locations considered in this study, but for a different study, and are thus not presented here.

### Herbivorous insects

Insects were assessed from 227 samples of 109 tree species, collected at 31 locations and in 18 countries (Fig. 1, Table 1).

The collected twigs (twenty 50 cm twigs per species per location) were brought to a laboratory close to each sampling location and inspected for the presence of insects that overwinter as adults. Twigs were kept at room temperature with the cut ends immersed in water to induce budding and to allow the development of insects that overwinter as larvae, pupae or eggs. Twigs from each sample were protected with gauze bags to prevent insects moving between samples (Fig. 1). Twigs were inspected for the presence of insects daily for 4 weeks and all collected insects were stored in 95% ethanol for further examination.

#### Morphological and molecular identification

Insects were inspected using a stereo microscope and sorted to taxonomic orders and feeding guilds (i.e. herbivores, predators, parasitoids and other). The abundance of the different feeding guilds and taxonomic orders in the samples is presented in a file deposited on Figshare^24^. Herbivorous insects were further sorted into morphospecies and at least one specimen per morphospecies was stored at −20 °C for molecular analysis. The abundance of the different morphospecies in each sample is presented in a file deposited on Figshare^24^. Specimens for molecular analysis were photographed with a Leica DVM6 digital microscope and the Leica Application Suite X (LAS X). Depending on the size of the insects, the whole individual or parts (e.g. legs, head) were used for molecular analysis. Genomic DNA was extracted with a KingFisher (Thermo Fisher Scientific) extraction protocol suitable for insects (35 min incubation at RT, 30 min wash at RT with 3 different washing buffers, 13 min elution at 60 °C) in a 96-well plate. PCR for the COI was carried out in 25 µl reaction volume with 2 µl diluted DNA (1:10), 0.5 µM of each of the primers LCO1490 and HCO2198^33^ and 1 x REDTaq ReadyMix Reaction Mix (Sigma-Aldrich) using a Veriti 96-Well Thermal Cycler (Applied Biosystems) with the following setting: 2 min at 94 °C, five cycles of 30 s at 94 °C, 40 s at 45 °C, and 1 min at 72 °C, 35 cycles of 30 s at 94 °C, 50 s at 51 °C, and 1 min at 72 °C, and a final extension step at 72 °C for 10 min. The success of amplification was verified by electrophoresis of the PCR products in 1.5% (w/v) agarose gel at 90 V for 30 min with ethidium bromide staining. A standard Sanger sequencing of the PCR products in both directions with the same primers was done at Macrogen Europe, Amsterdam, Netherlands. Sequences were assembled and edited with CLC Workbench (Version 7.6.2, Quiagen) and compared to reference sequences in BOLD^34^. If no conclusive results were found, sequences were compared to reference sequences in the National Centre for Biotechnology Information (NCBI) GenBank databases^35^. Specimens were assigned to species if the query sequence showed less than 1% divergence from the reference sequence. If two or more taxa matched within the same range, the assignment was ranked down to the next taxonomic level (i.e., genus). When no species match was obtained based on the above criteria, a genus was assigned with a divergence of less than 3%. For lower taxonomic groups the 100 nearest sequences were inspected on the Blast Tree (Fast Minimum Evolution Method) and the taxonomic relationship was evaluated based on that tree. If none of the approaches above revealed a conclusive taxonomic assignment, the morphological identification was taken as reference. The results of morphological and molecular identification of insect specimens are presented in a file deposited on Figshare^24^. Insect sequences are deposited in GenBank database under accession numbers MW441337-MW441767^25^.

### Sample metadata

Pairwise geographic distances (Euclidean distances) between sampling locations were calculated based on the geographic coordinates of the locations, with function “dist” in the R statistical programme^36^.

Climate data, including mean annual temperature, mean annual precipitation, and temperature seasonality were obtained from the WorldClim database^37^, at a resolution of 2.5 min, and represent averages between 1970 and 2000.

A host-tree phylogeny was constructed with the phylomatic function from the package brranching^38^ in R using the “zanne2014” reference tree^39^. One *Eucalyptus* sample collected in Argentina and two *Eucalyptus* samples collected in Tunisia were not identified to species. To place them in the phylogeny, we assigned them to different congeneric species that were not sampled in this study and that we considered as representative samples of phylogenetic diversity from across *Eucalyptus* genus (*E. viminalis*, *E. robusta* and *E. radiata*). Pairwise phylogenetic distances between study tree species were calculated using the “cophenetic” function in R^36^.

The described sample metadata are available in a file on Figshare^24^.

## Data Records

The raw paired-end Illumina sequencing reads of the ITS2 region obtained from fungal DNA extracted from pooled twig tissues are archived at the NCBI Sequence Read Archive under BioProject accession number PRJNA708148^22^. For each of 352 samples, two fastaq files are deposited, corresponding to reverse and forward sequence reads.

The excel file “Sample x fungal Amplicon Sequence Variant matrix” shows the distribution of 11,613,187 sequence reads of 12,721 fungal ASVs among 352 samples that were used for the fungal assessment and is available on Figshare^24^.

An Excel table with the identity of obtained fungal ASVs (i.e., “Identity of fungal Amplicon Sequence Variants”) is also available on Figshare^24^. The ASVs belonged to six phyla, with the majority belonging to the Ascomycota (68%) and the Basidiomycota (15%). Around 17% of ASVs could not be assigned to a phylum. Furthermore, 9,150 ASVs (72%) were assigned to one of 28 identified classes. The five most numerous classes were: Dothideomycetes (26%), Sordariomycetes (9%), Eurotiomycetes (9%), Leotiomycetes (7%), Tremellomycetes (6%). Only 31% of the ASVs were assigned to one of 661 identified genera, and 14% to one of 760 species.

A total of 4,751 insect specimens were reared from 227 samples. The number of insects in different feeding guilds and orders is provided for each sample from which insects were collected (i.e. 154 out of 227 samples) in the excel file “Insect specimens per feeding guild, taxonomic order and sample” and is stored on Figshare^24^. Almost 64% of collected specimens were classified as herbivores (3,032), around 10% were classified as parasitoids (499) and only 2% were predators (89). An additional 1,131 out of 4,751 (24%) specimens could not be assigned to a feeding guild.

An excel file showing abundances of 3,032 specimens of herbivorous insects, belonging to 208 morphospecies, in 227 collected samples (i.e., “Sample x herbivorous insect morphospecies matrix”) is provided on Figshare^24^. Herbivorous insects were from seven orders: Hemiptera (78.0%), Hymenoptera (11.9%), Lepidoptera (7.1%), Coleoptera (1.3%), Diptera (0.9%), Thysanoptera (0.7%) and Orthoptera (0.1%). A table containing data on the tentative identification of representative specimens of herbivorous insect morphospecies^24^ is also provided. A total of 554 specimens were sequenced and 431 yielded good quality sequences. Of those, 206 sequences were assigned to species, and an additional 123 were assigned to a genus. Assembled herbivorous insect COI sequences are deposited in GenBank database under accession numbers MW441337-MW441767^25^.

Data records on host identity (i.e. host species, hemisphere of origin and native or non-native host distribution range), geographic location (i.e. country of collection, location, latitude and longitude) and climate (i.e. mean annual temperature, temperature seasonality, mean annual precipitation) are also provided for each sample in the excel file “Sample Metadata” on Figshare^24^.

## Technical Validation

Sampling and sample processing were done in a standardized way as described above. All samples that were not collected and processed following the protocols were discarded (33 fungal samples). Negative controls were used for DNA extractions and PCRs to ensure the absence of external and cross contamination, and if controls were positive, samples were not used (21 fungal sample). Positive PCR controls were used to ensure that PCRs were performed correctly.

## Data Availability

R functions and databases used for generating the sample metadata are specified in the method section. A customized pipeline used for quality filtering of the raw sequence data obtained from HTS, delineation into ASVs and taxonomic classification of ASVs as described in Herzog *et al*.^30^ is available as a “ITS2.bash” file from the Zenodo repository^28^.
